# Microwave Irradiation Pre-Treatment as a Sustainable Method to Obtain Bioactive Hydrolysates from Chicken Feathers

**DOI:** 10.3390/ijms26136344

**Published:** 2025-06-30

**Authors:** Álvaro Torices-Hernández, Marta Gallego, Leticia Mora, Fidel Toldrá

**Affiliations:** Instituto de Agroquimica y Tecnologia de Alimentos (IATA-CSIC), Avenue Agustin Escardino 7, 46980 Paterna, Spain; atorices@iata.csic.es (Á.T.-H.); mgallego@iata.csic.es (M.G.); lemoso@iata.csic.es (L.M.)

**Keywords:** chicken by-products, enzymatic hydrolysis, bioactive peptides, microwave irradiation pre-treatment, antioxidant, antidiabetic, antihypertensive

## Abstract

Chicken feathers constitute a major by-product from the poultry industry, with a potential environmental impact and significant difficulties in their management. This study aimed to develop a sustainable method to hydrolyse chicken feathers and evaluate the effects of microwave (MW) irradiation pre-treatment in the generation of bioactive hydrolysates by simple or sequential hydrolysis with Alcalase. The hydrolysate with MW irradiation pre-treatment and Alcalase (2%, 2 h) (MWA) showed the highest overall antioxidant activity and neprilysin-inhibitory activity (55%), whereas samples without MW irradiation pre-treatment exerted the highest inhibitory activity of dipeptidyl peptidase IV (DPP IV) and angiotensin-converting enzyme (ACE-I), with values close to 50 and 70%, respectively. Mass spectrometry in tandem of bioactive hydrolysates was performed, and an in silico approach was used to characterise the obtained sequences. These results confirmed that MW irradiation pre-treatment improved Alcalase hydrolysis, leading to the generation of bioactive peptides with potential multifunctional properties, including antioxidant, antidiabetic, and antihypertensive activities. Moreover, this study highlights the potential of combining MW irradiation and enzymatic hydrolysis as a sustainable strategy for the revalorisation of chicken feathers.

## 1. Introduction

Over the past 10 years, the poultry industry has increased its production by more than 30% due to the rise in global population, with total production reaching 140 million of tons of poultry meat in 2022 [1]. Consequently, near 5 million tons of chicken feathers are generated every year from the poultry industry [2]. Part of this waste is used to obtain flours for animal feed, whereas the majority is landfilled, causing soil and water pollution [3,4,5]. In this regard, there is increasing interest in the revalorisation of feather waste in order to improve the negative economic and environmental impact.

The high protein content (>80%) in chicken feathers makes them attractive to obtain peptide rich hydrolysates, with a wide variety of applications such as plant fertiliser, as an ingredient in animal feed, in pharmaceuticals, and in cosmetics [6,7]. However, feathers are difficult to hydrolyse due to their high keratin content (>90%) [2], which is insoluble and highly resistant to hydrolysis [8]. So, identifying different sustainable treatments to extract and hydrolyse keratin protein are key to handling waste feathers.

Different chemical, physical, and biological (microbial and enzymatic) treatments have been reported to be successfully hydrolyse keratin, although not all of them are sustainable. Enzymatic hydrolysis was the most suitable for the obtention of bioactive peptides [3,9]. Chemical approaches are highly efficient in performing keratin hydrolysis, but they can cause the modification or degradation of amino acids and peptides, as well as generate contaminant residues, so these limitations mean chemical treatment is not recommended to obtain bioactive peptides [9]. Hydrothermal methods can lead to losses in thermolabile amino acids and the formation of potential toxic compounds, such as lysinoalanine and lanthionine, due to high temperatures [10]. Enzymatic hydrolysis needs to be performed under controlled and optimised conditions and presents high specificity, which makes it very convenient, but it is ultimately expensive due to the use of commercial enzymes and reactors, and the frequent low yield of obtained peptides [11]. Thus, a combination of physical, chemical, and biological treatments could be an interesting approach to overcome these challenges.

The use of microwave (MW) irradiation to accelerate chemical or enzymatic reactions has been explored in different food matrices. This process can lead to the denaturation of protein structures by facilitating subsequent hydrolysis, as well as improving the solubility, stability, and bioavailability of peptides [12]. MW irradiation in an alkali medium enhances the disruption of disulphide bonds and improves the chicken feather solubilisation compared to water bath heating treatment [13]. So, MW-assisted hydrolysis has been used to obtain protein hydrolysates rich in bioactive peptides from different sources, such as cricket [14], trout [15], soybeans [16], and sturgeon [17]. Santos et al. [18] studied the influence of using MW irradiation at 1400 W and enzymatic hydrolysis with Alcalase and Flavourzyme to obtain chicken feather hydrolysates, evaluating their antioxidant and technological properties. However, the use of high MW power could lead to protein denaturation due to the temperature increase in the sample. The application of gentler pre-treatments as well as sequential enzymatic hydrolysis could help to obtain keratin hydrolysates rich in bioactive peptides. In addition, it is necessary to perform the complete characterisation of the bioactive hydrolysates using last-generation peptidomic approaches to fully understand their potential influence over the different metabolisms as well as improve the knowledge of peptide generation in enzymatic hydrolysis. Therefore, the aim of this study was to evaluate the effect of using mild MW irradiation pre-treatment on chicken feathers hydrolysed with Alcalase, added simply or sequentially, to obtain a sustainable method able to hydrolyse chicken feathers by-products and obtain hydrolysates rich in bioactive peptides. The antioxidant, antidiabetic, and antihypertensive potential of the hydrolysates was evaluated, and chromatographic, mass spectrometry, and in silico analyses were used for the identification and quantification of the peptides in order to know their characteristics and establish a relationship with their bioactivity.

## 2. Results and Discussion

### 2.1. Chicken Feather Characterisation

The chemical composition of chicken feathers indicates a protein content of 76.71 ± 1.21%, whereas smaller amounts of fat (6.97 ± 0.97%), moisture (4.55 ± 0.22%), total carbohydrates (1.04 ± 0.2%), and ash (0.4 ± 0.02%) were found. These results are similar to those reported by Tesfaye et al. [6] and confirm the potential of chicken feathers as a good source of proteins to prepare hydrolysates.

**Figure 1:**
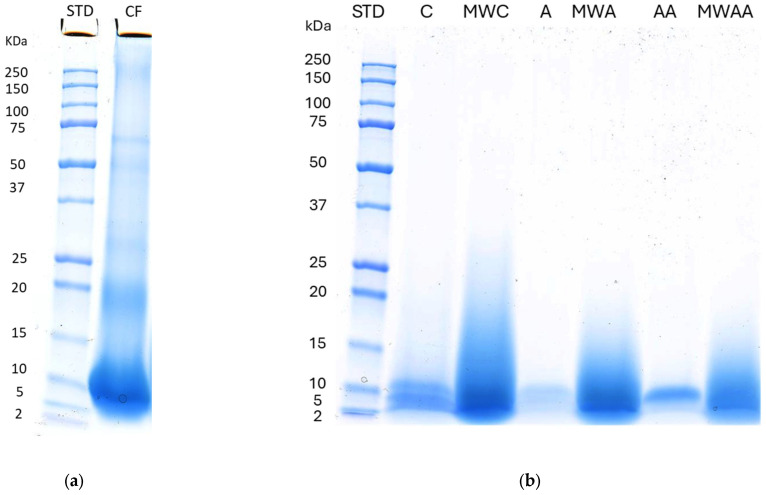
(**a**) SDS-PAGE of chicken feather as control, (**b**) SDS-PAGE profile of the deproteinised samples. (STD: protein standard; CF: chicken feather control; C: control, without microwave irradiation pre-treatment or enzymatic hydrolysis; MWC: microwave irradiation pre-treatment control, without enzymatic hydrolysis; A: simple Alcalase hydrolysis; MWA: microwave irradiation pre-treatment and simple Alcalase hydrolysis; AA: sequential Alcalase hydrolysis; MWAA: microwave irradiation pre-treatment and sequential Alcalase hydrolysis).

Chicken feathers were subjected to simple or sequential hydrolysis with Alcalase, preceded or not by MW irradiation treatment. The protein profiles of the different samples were characterised by Sodium Dodecyl Sulphate Polyacrylamide Gel Electrophoresis (SDS-PAGE) (Figure 1). Figure 1a shows the SDS-PAGE of chicken feather control (C) without deproteinisation, whereas small bands in the ranges of 50–75 kDa and 2–20 kDa, and a major band between 5 and 10 kDa were found, indicating the presence of α-keratin (40–70 kDa) and β-keratin (10–22 kDa) [19]. A gel image of deproteinised samples (Figure 1b) shows bands between 2 and 10 kDa in A and AA samples (simple and sequential Alcalase hydrolysates, respectively), whereas the bands in MW irradiation-pre-treated samples hydrolysed by the simple or sequential addition of Alcalase (MWA and MWAA, respectively) were more intense and show protein fragments in a wider range of 2–17 kDa. These results indicate that MW irradiation pre-treatment could either facilitate the extraction of proteins or generate new intermediate and low-molecular-weight peptides.

Chicken feathers are highly difficult to hydrolyse due to the fact that keratin shows a high content of half cystine, forming disulfide linkages (intermolecular and intramolecular) and interchain peptide linkages [8], as well as a high serine content that can contribute to the formation of non-covalent interactions such as hydrogen bonds and hydrophobic and electrostatic forces [20]. However, the obtained results suggest that the combination of both MW irradiation and hydrolysis with Alcalase would be a successful approach to hydrolyse chicken feathers. Škerget et al. [21] found small-molecular-weight protein fragments (4–12 kDa) when the chicken feathers were hydrolysed with subcritical water at high temperatures. Khumalo et al. [22] also observed protein fragments of low molecular weight (10–15 kDa) after the extraction of keratin with reducing agents from chicken feathers. Other studies observed that MW irradiation pre-treatment can enhance the gastrointestinal digestion of quinoa protein, increasing the degradation of the proteins in smaller proteins fragments (<10 kDa) and improving the digestibility [23].

### 2.2. Effects of Microwave Irradiation Pre-Treatment and Enzymatic Hydrolysis on the Bioactivity of Samples

The potential antioxidant, antihypertensive, and antidiabetic activities of the chicken feather samples were evaluated to assess the impact of the MW irradiation pre-treatment and enzymatic hydrolysis on the bioactivity.

**Figure 2:**
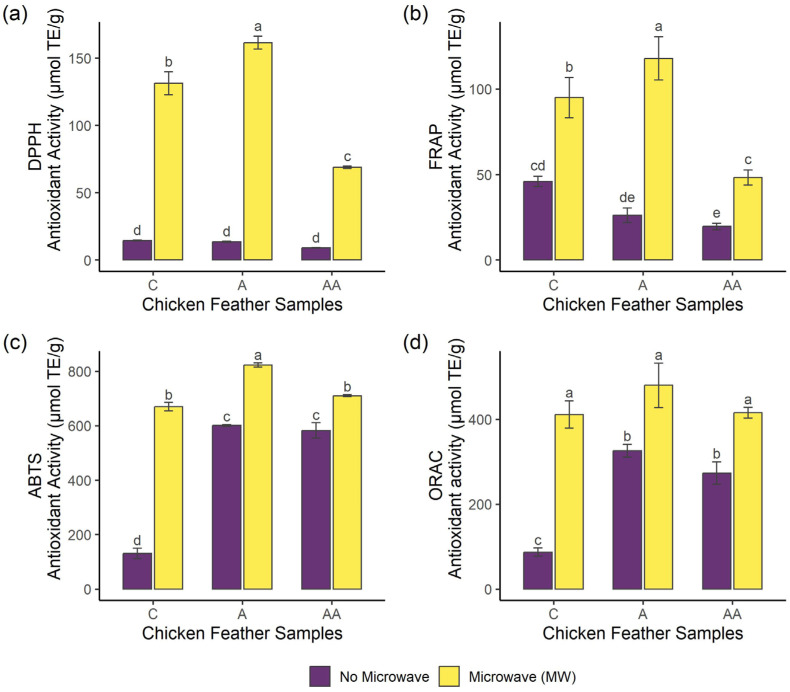
Antioxidant activities of the deproteinised samples measured by (**a**) DPPH, (**b**) FRAP, (**c**) ABTS, and (**d**) ORAC. Each test was performed in triplicate (*n* = 3). Different letters show significant differences (*p* < 0.05). (C: control, without enzymatic hydrolysis; A: simple Alcalase hydrolysis; AA: sequential Alcalase hydrolysis).

The overall antioxidant capacity of the samples was evaluated using four different methods, including DPPH radical scavenging activity (DPPH), ferric-reducing antioxidant power (FRAP), ABTS radical scavenging capacity (ABTS), and oxygen radical absorbance capacity (ORAC). The use of more than one method is required to have a global view of the antioxidant capacity of a sample due to the different mechanisms of action of oxidative reactions [24]. The antioxidant results of the samples are shown in Figure 2. Regarding DPPH and FRAP methods, pre-treated samples reached higher antioxidant activity compared to non-pre-treated samples, where MWA hydrolysate showed the highest values (161.38 and 117.84 µmol TE/g in DPPH and FRAP, respectively (*p* < 0.05)), followed by MWC sample. On the other hand, MWAA exerted the lowest antioxidant activity (68.88 and 48.23 µmol TE/g in DPPH and FRAP, respectively) among pre-treated hydrolysates (*p* < 0.05). Concerning ABTS method, control without pre-treatment showed the lowest antioxidant activity (130.51 µmol TE/g), while samples treated with Alcalase (A and AA) showed higher antioxidant values. MW irradiation pre-treatment improved the antioxidant activity, where MWA reached the highest value in this assay (669.19 µmol TE/g), being statistically significant (*p* < 0.05). Regarding ORAC method, pre-treated samples achieved higher antioxidant values than non-pre-treated samples, although there were no significant differences between MW irradiation-pre-treated hydrolysates (*p* > 0.05). Therefore, the application of MW irradiation would increase the overall antioxidant activity of chicken feather samples, mainly when followed by simple enzymatic hydrolysis with Alcalase. Antioxidant properties of peptides depend on their hydrophobicity, structure, and composition [25]. In this regard, MW irradiation could lead to changes in protein structure [26,27] improving the accession of Alcalase to the cleavage sites and resulting in the generation of peptides with higher antioxidant activity. Performing sequential hydrolysis with Alcalase and MW irradiation resulted in a decrease in the antioxidant activity, probably due either to the generation of less active sequences [28] or to the formation of peptide complexes involving electrostatic and hydrophobic interactions [29]. Previous studies have reported the antioxidant activity of chicken feather hydrolysates obtained using different enzymes such as Savinase, Alcalase, and Flavourzyme, and different pre-treatments such as ultrasound and heating with NaOH [30,31,32,33,34]. Furthermore, Santos et al. [18] reported similar results on chicken feathers pre-treated with MW irradiation at 1400 W, and hydrolysed with Alcalase and Flavourzyme, showing an increase in DPPH and ABTS activity when MW irradiation was applied. In addition, MW irradiation pre-treatment was reported to enhance the antioxidant activity of fish protein hydrolysate of *Labeo rohita* by-products [35]. MW irradiation pre-treatment combined with ultrasound improved the antioxidant activity of *Acipenser sinensis* hydrolysates [17]. Li et al. [36] performed a combined hydrolysis with ionic liquids and the MW irradiation of different keratin-rich sources such as horse hair, rabbit hair, and chicken feather, resulting in high antioxidant activity values (86–99% DPPH scavenging rate).

**Figure 3:**
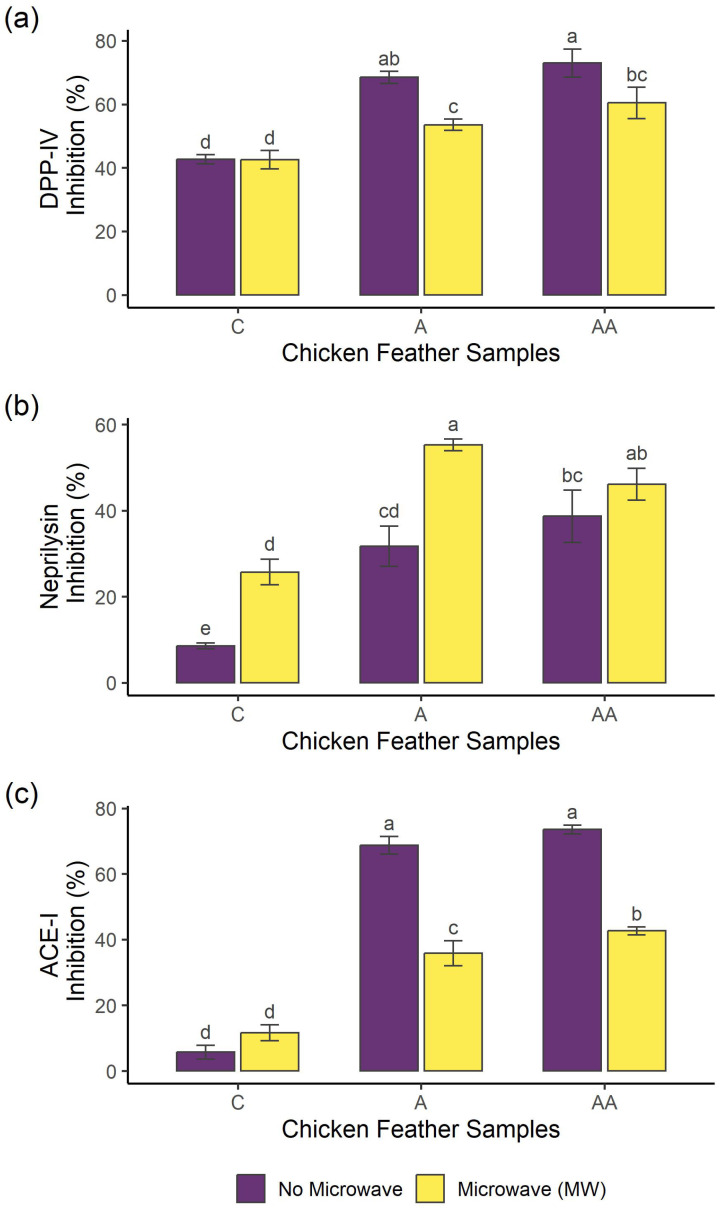
(**a**) DPP-IV enzyme inhibition activity, (**b**) Neprilysin enzyme inhibition activity, and (**c**) ACE-I enzyme inhibition activity of the deproteinised samples. Each test was performed in triplicate (*n* = 3). Different letters show significant differences (*p* < 0.05). (C: control, without enzymatic hydrolysis; A: simple Alcalase hydrolysis; AA: sequential Alcalase hydrolysis).

Inhibitors of the dipeptidyl peptidase IV (DPP-IV) enzyme increase the average life of glucagon-like peptide 1 (GLP-1) and glucose-dependent insulinotropic peptide (GIP). These incretins are rapidly degraded by DPP-IV enzyme, stimulating insulin secretion and thus being commonly used to treat type 2 diabetes [37]. The DPP-IV enzyme-inhibitory activity of the samples is shown in Figure 3a. Samples A and AA achieved the highest inhibition values (68 and 73%, respectively) showing no significant difference between both samples (*p* > 0.05), and the use of MW irradiation resulted in a decrease in this biological activity. These results suggest the potential of chicken feather hydrolysates to inhibit DPP-IV enzyme, which was previously predicted in silico in hydrolysates obtained with papain and subtilisin by using the BIOPEP-UWM software program [38]. The potential inhibitory action of the DPP-IV enzyme has been previously reported when using bacteria mediums on chicken feathers as a substrate [39]. Moreover, hydrolysates obtained with Alcalase from chicken by-products have shown DPP-IV inhibition [40]. MW irradiation pre-treatment was also applied in trout [15] and cricket [14], before the hydrolysis with Alcalase, obtaining potential DPP-IV-inhibitory hydrolysates.

Neprilysin is an endopeptidase that plays a role in glucose metabolism [41] and cardiovascular diseases like heart failure [42]. This enzyme has a wide range of substrates such as GLP-1 and vasodilator peptides, including bradykinin, substance P, and natriuretic peptides [41,42]. A combination of DPP-IV and neprilysin inhibitors could be an interesting approach for treating type 2 diabetes because they may have additive effects [41]. The neprilysin-inhibitory activity of the samples is presented in Figure 3b. MWA and MWAA showed the best values among the hydrolysates (55 and 47%, respectively) showing no significant difference between both hydrolysates (*p* > 0.05), whereas MW irradiation pre-treatment improved the inhibitory activity of sample A. Neprilysin peptide inhibitors derived from food or food by-products are scarcely investigated; however, recently, enzymatic hydrolysates obtained from meat by-products such as dry-cured ham bones, beef liver, and chicken carcass have been described to show neprilysin-inhibitory activity [43,44,45].

Angiotensin I-converting enzyme (ACE-I) is the enzyme responsible to cleavage angiotensin-I into angiotensin-II, which is a vasoconstrictor that can bind to different receptors, increasing blood pressure. Therefore, the inhibition of ACE-I leads to vasodilation effects, being used for the treatment of hypertension and inflammation-associated cardiovascular diseases [46]. The ACE-I-inhibitory activity of the samples is shown in Figure 3c. Samples without MW irradiation pre-treatment (A and AA) showed the highest inhibitory activity among the hydrolysates (69–74%, respectively), where no significant differences were found between these samples (*p* > 0.05), while the pre-treated hydrolysates (MWA and MWAA) showed lower activity values (35–42%, respectively). MW irradiation pre-treatment could modify the structure of proteins, resulting, after hydrolysis, in the release of peptides with characteristics less suitable for inhibiting this enzyme. Chicken feathers have been previously reported to have ACE-I-inhibitory activity when hydrolysed by different enzymes such as Alcalase and keratinase and subjected to different pre-treatments such as ultrasounds [4,32] and microbial processing [39]. However, few studies have investigated the application of MW irradiation as a treatment to enhance ACE-I-inhibitory activity. Hall & Liceaga [14] observed increased ACE-I inhibition in cricket hydrolysates obtained with Alcalase when applying MW irradiation as a pre-treatment compared to a water bath, obtaining IC_50_ values of 0.096 mg/mL and 0.20 mg/mL, respectively. MW-assisted hydrolysis on black soybean also showed a 12% increment of ACE-I-inhibitory activity [16].

### 2.3. Peptidomics Characterisation of the Samples

Hydrolysates A and MWA, which showed the highest overall bioactivity, as well as control samples (C and MWC), were analysed by liquid chromatography-tandem mass spectrometry (LC-MS/MS) to identify the sequences of the generated peptides. Supplementary Table S1 shows the list of the peptides identified in each sample (C, MWC, A and MWA). A total of 1,675 unique peptide sequences were found in C, while 1548 peptides were identified in the control when MW irradiation was applied. On the other hand, the enzymatic treatment with Alcalase increased the release of peptides, since 10,687 and 13,157 peptide sequences were found in the hydrolysates A and MWA, respectively. Therefore, MW irradiation pre-treatment facilitated the release of more unique peptide sequences after enzymatic hydrolysis.

**Figure 4:**
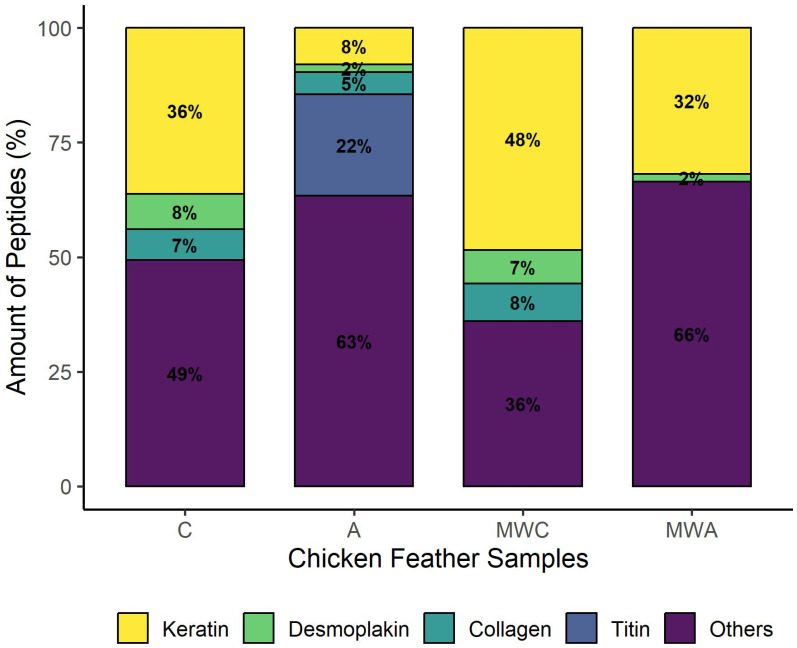
Protein distribution of unique peptides identified in each sample. (C: control, without microwave irradiation pre-treatment or enzymatic hydrolysis; A: simple Alcalase hydrolysis; MWC: microwave irradiation pre-treatment control; without enzymatic hydrolysis; MWA: microwave irradiation pre-treatment and simple Alcalase hydrolysis).

Figure 4 shows the distribution of the identified peptides in each sample according to their protein of origin. Keratin, collagen, and desmoplakin were the main parent proteins of the peptides identified in control samples, whereas MW irradiation increased the number of peptides derived from keratin from 36% in C to 48% in MWC. Keratin (8%) and titin (22%) were the main origin proteins of peptides identified in sample A, whereas the number of peptides derived from keratin increased in MWA up to 32%. MW irradiation in an alkali medium can lead to structural changes in keratin, modifying amide A and amide I and causing disruption in hydrogen bonds (amide A) to give free N-H and in the β-sheet structure (amide I) [13]. This could facilitate enzymatic hydrolysis by Alcalase, incrementing the release of peptides from keratin when MW irradiation was applied as pre-treatment.

The identified peptides were analysed using in silico tools, and the results are shown in Table 1. Peptides with a high probability to be bioactive, with a PeptideRanker Ratio (PRR) > 0.97, were characterised and their potential bioactivities according to BIOPEP database were indicated. A total of 31 peptide sequences were identified. The range was between 8 and 26 residues. Concerning the potential of the identified peptides, MWA showed the highest number of peptides with PRR > 0.97. Among the peptides with the highest A values, which indicate the presence of possible bioactive fragments in the sequence, FGGGGFGGGGFGGGFGG (identified in sample MWC) showed high potential as an ACE and neprilysin inhibitor, PPGPPGPPGPPSGGF (sample A) as a DPP-IV inhibitor, and GAAFMLGF (sample A) as an antioxidant. All the peptides have hydrophobicity values ≤ 0.35. Potential peptide fragments predicted as bioactive are also indicated (Table 1). DPP-IV-inhibitory peptides are reported to range from 3 to 13 amino acids, and most of them have hydrophobic residues [47]. Moreover, hydrophobic amino acids at C-terminal and next to N-terminal contribute to the inhibition of DDP-IV [48]. Hydrophobic interactions between the inhibitor and the catalytic domain of the enzyme are also suggested to enhance inhibition by the presence of amino acids with aromatic rings [49]. Peptides composed of arginine, valine, proline, alanine, and glutamic acid are likely to be neprilysin inhibitors, while those with alanine in the N-terminal position and glutamic acid or arginine in the C-terminal may favour the inhibition of the enzyme [43]. Peptides can exert the inhibition of ACE-I based on certain properties, such as specific aliphatic amino acids like valine, leucine, isoleucine and glycine in N-terminal, and aromatic amino acids like tyrosine, tryptophan, and proline at the C-terminal [50]. ACE-I-inhibitory peptides are usually short-chain peptides (2–12 amino acids in length) and those composed by 4 or more residues usually contain basic amino acids (lysine and arginine) at the C-terminal [50,51]. Also, these peptides are characterised for having hydrophobic, acidic, and positively charged residues in their sequences [52].

Dipeptides and tripeptides are usually of high interest because their potential bioactivity, including inhibition of ACE-I, DPP-IV, and Neprilysin. Dipeptides GG, GF, and FG were the most predominant residues among the analysed sequences. In this regard, GG and GF were previously reported as in vitro DPP-IV inhibitors [53], whereas GG and GP, derived from chicken carcass hydrolysates, were recently reported as in vitro Neprilysin inhibitors [43].

**Figure 5:**
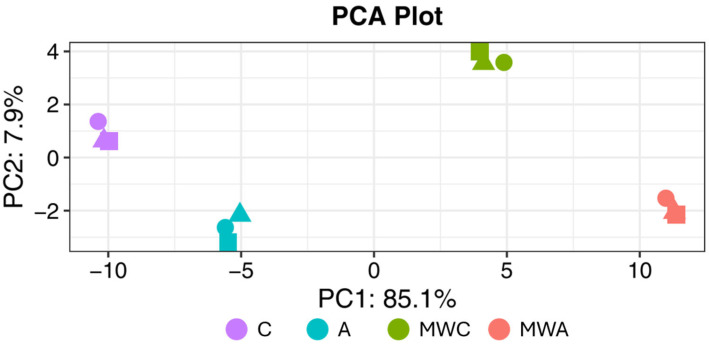
Principal Component Analysis plot of samples processed under different conditions. (C: control, without microwave irradiation pre-treatment or enzymatic hydrolysis; A: simple Alcalase hydrolysis; MWC: microwave irradiation pre-treatment control, without enzymatic hydrolysis; MWA: microwave irradiation pre-treatment and simple Alcalase hydrolysis).

**Figure 6:**
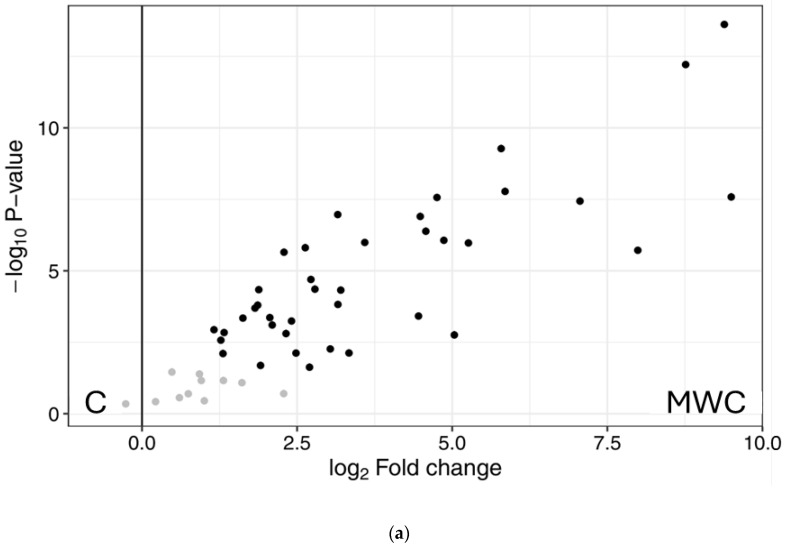

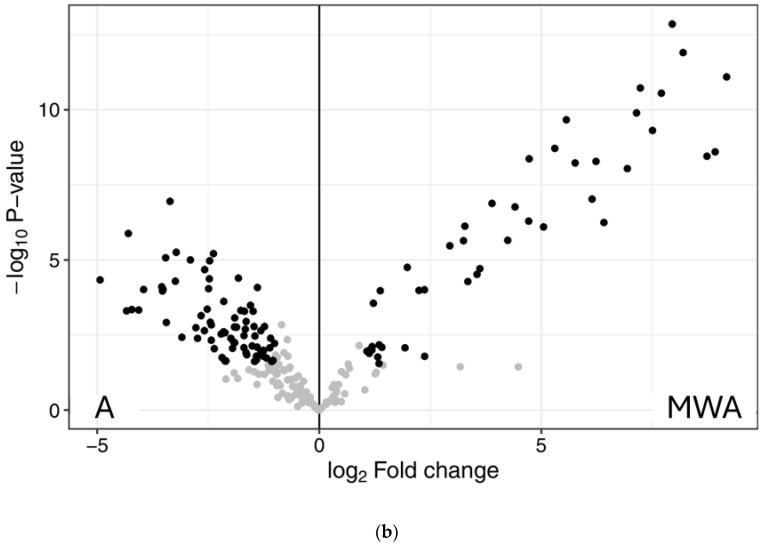
Volcano plots of samples (**a**) C versus MWC and (**b**) A versus MWA. Different colour shows significant differences (*p* < 0.05). (C: control, without microwave irradiation pre-treatment or enzymatic hydrolysis; MWC: microwave irradiation pre-treatment control, without enzymatic hydrolysis; A: simple Alcalase hydrolysis; MWA: microwave irradiation pre-treatment and simple Alcalase hydrolysis).

A total of 2823 peptide sequences were relatively quantified, obtaining a total of 239 peptides that differed significantly between samples. Figure 5 shows the Principal Component Analysis (PCA) plot of the samples, evidencing four different clusters. Principal Component 1 was able to explain 85.1% of the differences between the groups with and without MW irradiation pre-treatment, and Principal Component 2 explained the 7.9% of variance within the dataset. So, samples with no MW irradiation treatment were grouped on the left of the PCA plot, whereas both groups of samples subjected to MW irradiation were on the right. Figure S1 shows a plot representing an overview of expression of all significant (differentially expressed) peptide sequences (rows) in all samples (columns), showing a high reproducibility between triplicates. In addition, volcano plots were represented (Figure 6), so each point on the graph represents a peptide. The y axis shows the level of statistically significant differences in protein abundance, whereas the x axis represents the magnitude of the change. In this sense, Figure 6a shows the effect of MW irradiation treatment on control samples, showing a significant number of peptides with higher abundance values in MWC than in C. On the other hand, Figure 6b compares samples MWA versus A, showing that, although there is a higher number of less abundant peptides in A, some of the identified peptides showing the highest abundancy in MWA were also the ones showing greater significant differences between samples. In fact, the higher number of less abundant peptides in A could be due to a lower number of peptides identified in A versus MWA due to the effect of MW irradiation treatment.

## 3. Materials and Methods

### 3.1. Chemicals and Reagents

Chicken feathers were from *Hy-Line* brown laying hens, supplied from a local farmer (Castellón). Alcalase^®^ 2.4 L was purchased from Novozymes A/S (Bagsvaerd, Denmark). Phenol, (±)-6-hydroxy-2,5,7,8-tetramethylchromane-2 carboxylic acid (Trolox), 2,2-diphenyl-1-picrylhydrazyl (DPPH), potassium ferricyanide, ferric chloride, 2,2-azino-bis(3-ethylbenzothiazoline-6-sulfonic acid) diammonium salt (ABTS), ascorbic acid, fluorescein, 2,2-azobis(2-methylpropionamidine) dihydrochloride (AAPH), L-tryptophan, N-Succinyl-Ala-Ala-Phe-7-amido-4-methylcoumarin (AMC), HEPES, thiorphan, angiotensin I-converting enzyme (ACE-I) from rabbit lung, and captopril were purchased from Sigma-Aldrich, Co. (St. Louis, MO, USA). Aminopeptidase M was purchased from Merck (Darmstadt, Germany). *o*-Aminobenzoylglycyl-*p*-nitro-*L*-phenylalanyl-*L*-proline (Abz-Gly-Phe(NO2)-Pro) trifluoroacetate salt was purchased from Bachem AG. (Bubendorf, Switzerland), whereas butylated hydroxytoluene (BHT), potassium persulfate, and D(+)-sucrose were purchased from Panreac Química, S.A.U. (Barcelona, Spain). Human Neprilysin Protein Tag free was obtained from Acro BioSystems (Basel, Switzerland) and DPP-IV (Dipeptidyl Peptidase-IV) inhibitor screening kit was obtained from Abcam Plc. (Cambridge, UK). All other chemicals and reagents were of analytical grade.

### 3.2. Obtention of Chicken Feather Samples

#### 3.2.1. Preparation of Chicken Feather Meal

The feather meal was prepared according to the methodology described by Cheong et al. [54] with modifications. The feathers were washed thoroughly with distilled water at 60 °C, dried at 40 °C for 2 days in an oven, and then cut with scissors and freeze-dried. Finally, the feathers were milled in a Mixer Mill MM400 (Retsch GmbH, Haan, Germany), at a frequency of 25 kHz for 5 min. The chicken feather meal was stored in a desiccator until further analysis.

#### 3.2.2. Chemical Composition of Chicken Feathers

Protein and peptide content was determined by Dumas combustion according to the AOAC method 992.15 [55]. Fat content was determined by the method of Folch et al. [56], whereas the moisture of the feathers was determined by drying them in a halogen moisture analyser (HB43, Mettler Toledo, Greifensee, Switzerland). Total carbohydrate content was determined by the phenol-sulphuric acid method described by Quero-Jiménez et al. [57]. Ash content was calculated after the overnight ignition of the samples at 600 °C, as described by Tesfaye et al. [6].

#### 3.2.3. Microwave Irradiation Pre-Treatment and Enzymatic Hydrolysis

**Figure 7:**
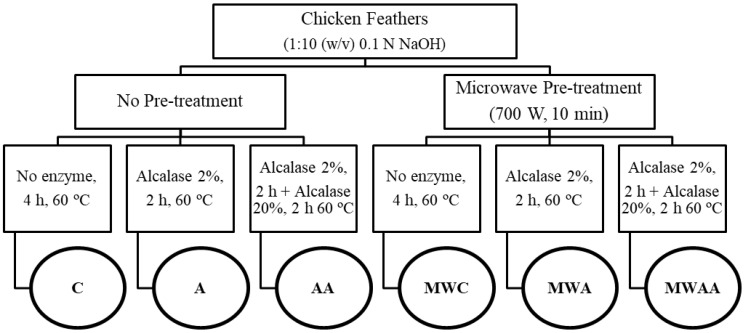
Scheme for obtaining the different chicken feather samples.

Chicken feather hydrolysates were obtained by mixing 2 g of the meal with 20 mL of 0.1 N NaOH, following the procedure shown in Figure 7. Two groups of samples were prepared: one was not pre-treated with microwave (MW) irradiation, and the second group was summited to MW irradiation pre-treatment. For that, samples were heated in a domestic microwave oven (ProClean 3010, Cecotec, Valencia, Spain) at 700 W for 10 min, pausing every 20 s and cooling the samples in a water/ice bath to avoid overheating (<70 °C). Then, both groups were summited to two different enzymatic hydrolysis (simple or sequential) in a ball reactor (Carousel 6 Plus Reaction Station, Radleys, Safron Walden, UK), using Alcalase enzyme and previously adjusting the pH to 8.0 with 6 M HCl. Simple enzymatic hydrolysis was performed at 60 °C for 2 h with Alcalase (2%, *w*/*w*), whereas sequential hydrolysis was performed by adding Alcalase at high concentration (20%, *w*/*w*) for additional 2 h. Control samples were prepared by applying or not MW irradiation pre-treatment and without enzyme addition. After hydrolysis, samples were heated at 90 °C for 10 min to inactivate the enzymes. The samples were then centrifuged at 22,000× *g* for 15 min, the supernatants were collected, and the pellets were dissolved with bidistilled water and centrifuged again under the same conditions. The centrifugation process was repeated three times to extract all generated peptides and obtain the maximum yield, and resulting supernatants from each sample were put together and freeze-dried. Finally, samples were deproteinised by adding 3 volumes of ethanol and maintaining at 4 °C overnight. After centrifugation at 12,000× *g* for 10 min at 4 °C, supernatants were collected, ethanol was removed, and the deproteinised samples were freeze-dried and stored at −20 °C for further analysis.

### 3.3. SDS-PAGE Electrophoresis

The protein profile of the samples was evaluated by SDS-PAGE electrophoresis, according to Laemmli [58] with some modifications. Each sample was resuspended in 1 mL of Laemmli Sample Buffer for 10 mg/mL, and then, denatured at 95 °C for 5 min. A total of 20 µL of each sample was loaded in the gel and the electrophoresis was performed using an AnyKD gel (BioRad: Cat# 4569034) at 200 V for 30 min. The gel was fixed with 40% ethanol/10% acetic acid for 1 h and stained with colloidal Coomassie (Bio-Rad Laboratories, Madrid, Spain) for 1 h. Finally, the gel was destained with bidistilled water and scanned with Image Scanner (GE Healthcare, Chicago, IL, USA). The results were analysed using the image processing and analysis software program ImageJ v. 1.52 (Wayne Rasband, National Institute of Mental Health, Washington, DC, USA) to obtain the protein profile image.

### 3.4. Antioxidant Activity

The antioxidant activity was evaluated by using four different methods as previously described by Gallego et al. [59]. All the results were expressed as µmol of Trolox Equivalents (TE) per gram of the deproteinised sample, calculated from standard curves of Trolox. Analyses were performed in triplicate.

#### 3.4.1. DPPH Radical Scavenging Activity

The DPPH method was performed by mixing 100 µL of each deproteinised sample (1–20 mg/mL) with 500 µL of ethanol 96% and 125 µL of DPPH reagent (0.02% in ethanol). The mixture was incubated for 1 h in the dark at room temperature. The absorbance was measured at 517 nm in a UV-vis spectrophotometer (Cary 60, Agilent Technologies, Santa Clara, CA, USA). BHT (2 mg/mL) was used as the positive control.

#### 3.4.2. Ferric-Reducing Antioxidant Power (FRAP)

FRAP assay was performed by mixing 80 µL of each deproteinised sample (5 mg/mL) with 80 µL of phosphate buffer (200 mM, pH 6.6) and 80 µL of potassium ferricyanide (10 mg/mL). The mixture was incubated at 50 °C for 20 min, and then 80 µL of trichloroacetic acid (100 mg/mL) was added to stop the reaction, followed by centrifugation at 200× *g* for 10 min. Finally, 140 µL of the supernatant was mixed with 140 µL of bidistilled water and 28 µL of ferric chloride (1 mg/mL). The mixture was kept in the dark for 10 min, and subsequently the absorbance was measured at 700 nm using a microplate reader (CLARIOstar Plus, BMG LABTECH GmbH, Ortenberg, Germany). BHT (20 mg/mL) was used as the positive control.

#### 3.4.3. ABTS Radical Scavenging Capacity

The ABTS method was performed by dissolving ABTS (7 mM) in potassium persulfate (2.45 mM) and kept in the dark overnight. This solution was diluted with phosphate-buffered saline (PBS) (50 mM, pH 7.4), until reaching an absorbance of 0.70 ± 0.02 at 734 nm, which was used as the ABTS working solution. A volume of 10 µL of each deproteinised sample (1 mg/mL) was mixed with 990 µL of the ABTS working solution, incubated for 6 min, and the absorbance was measured at 734 nm in the UV-vis spectrophotometer Cary 60. Ascorbic acid (4 mM) was used as the positive control.

#### 3.4.4. Oxygen Radical Absorbance Capacity Assay (ORAC)

An ORAC assay was performed by mixing 140 µL of the deproteinised sample (25 μ/mL) with 70 µL of fluorescein (200 mM) prepared in phosphate buffer (75 mM, pH 7.4). The mixture was incubated at 37 °C for 15 min, and then 70 µL of AAPH (80 mM) was added. The fluorescence was measured at intervals of 1 min for a total time of 100 min, using excitation and emission wavelengths of 485 nm and 538 nm, respectively, in the microplate reader CLARIOstar Plus. Tryptophan (3 µM) was used as the positive control.

### 3.5. DPP-IV-Inhibitory Activity

The DPP-IV inhibition was evaluated using the DPP-IV Inhibitor Screening Assay Kit (Cayman Chemical, Ann Arbor, MI, USA), following the protocol provided by the manufacturer. The assay uses the fluorogenic substrate, Gly-Pro-aminomethylcoumarin, so the cleavage of peptide bonds by the DPP-IV enzyme releases the free aminomethylcoumarin group, resulting in fluorescence. For the assay, 10 µL of the deproteinised sample (20 mg/mL) was mixed with 10 µL of DPP-IV, 30 µL of assay buffer, and 50 µL of substrate. The mixture was incubated at 37 °C for 30 min, and the fluorescence was measured using excitation and emission wavelengths of 360 nm and 460 nm, respectively. Sitagliptin (1 mM) was used as the positive control. Samples were assayed in triplicate.

### 3.6. Neprilysin-Inhibitory Activity

Neprilysin inhibition was determined following the method described by Moreno-Mariscal et al. [43]. Briefly, a stock enzyme solution was prepared at a concentration of 400 µg/mL in 0.1% bovine serine albumin/water and diluted with bidistilled water up to 400 ng/mL. AMC was used as the substrate, preparing a 0.2 mM stock solution in 50 mM HEPES/NaOH buffer at pH 7.4. The substrate solution was prepared by mixing the stock substrate solution with aminopeptidase M, considering the addition of 0.75 µg of aminopeptidase M/well. The assay was performed by mixing 25 µL of the deproteinised sample (1 mg/mL) and 25 µL of the enzyme solution, and after incubation at 37 °C for 10 min in dark, 50 µL of the substrate solution was added. The fluorescence was measured in the microplate reader CLARIOstar Plus, using excitation and emission wavelengths of 320 nm and 420 nm, respectively, in kinetic mode for 1 h at 37 °C. Thiorphan at 1 µM was used as the positive control. Samples were assayed in triplicate.

### 3.7. ACE-I-Inhibitory Activity

The ACE-I-inhibitory activity determines the capacity of ACE-I to hydrolyse the fluorescent substrate Abz-Gly-Phe-(NO_2_)-Pro. The activity was measured following the methodology described by Sentandreu and Toldrá [60]. A total volume of 50 µL of the deproteinised sample (1 mg/mL) was mixed with 50 µL of the enzyme (3 mU/mL ACE-I in 150 mM Tris base buffer, pH 8.3). To start the reaction, 200 µL of substrate (0.45 mM Abz-Gly-p-nitro-Phe-Pro-OH in 150 mM Tris base buffer with 1.125 mM NaCl pH 8.3) was added, and the mixture was incubated at 37 °C for 45 min. The fluorescence was measured at excitation and emission wavelengths of 355 nm and 205 nm, respectively. Captopril (10 µM) was used as the positive control. Samples were assayed in triplicate.

### 3.8. Peptidomic Characterisation of the Samples

#### 3.8.1. Peptide Analysis by LC-MS/MS

Identification of peptides was performed by LC-MS/MS using the EvoSep One LC system (Odense, Denmark) coupled to a Tims TOF fleX mass spectrometer (Bruker Daltonik GmbH, Bremen, Germany). Firstly, 1 µL of each sample (1 mg/mL) was quantified by nanoDrop (Thermo Scientific, Waltham, MA, USA) (A 280 nm, ε = 1 mg/mL). A total of 20 µL of the sample, in 0.1% formic acid (FA), was loaded in an Evotip pure tip (EvoSep) according to manufacturer instructions. The sample loaded was eluted to an analytical column (EvoSep 15 cm × 150 µm, 1.5 µm; Evosep) by the Evosep One LC system, and solved with the 30 SPD chromatographic method defined by the manufacturer. The eluted peptides were ionised in a captive spray with 1700 V at 200 °C and analysed using ddaPASEF mode with the following settings in TIMS: mode custom, 1/K0 0.7–1.76 V.s/cm^2^, ramp time of 100 ms, 100% duty cycle, and ramp rate of 9.42 Hz. The MS settings were as follows: scan between 100 and 1700 *m/z*, positive polarity ion mode, and scan mode PASEF. The MS/MS settings were as follows: 4 PASEF ramps, 0.5 s of total cycle time, 0–5 charges, target intensity of 12,500, intensity threshold of 1000, and active exclusion on. The system sensitivity was controlled with 20 ng of HELA digested proteins.

#### 3.8.2. Identification and Label-Free Quantification Data Analysis

The identification of non-tryptic peptides was performed using the Mascot Distiller software program 2.8 (Matrix Science, Inc., Boston, MA, USA) by filtering the UniProt protein database. The Mascot 2.8 search engine conditions were as follows: taxonomy: Chordata (vertebrates and relatives); enzyme: none; maximum missed cleavages: 2; peptide mass tolerance: 100 ppm; fragment mass tolerance: 0.3 Da.

In label-free quantitation data analysis, raw data files were analysed with FragPipe 23.0 using the IonQuant algorithm 1.11.9 to obtain protein identifications and label-free quantification values through the LFQ-MBR workflow of FragPipe. So, a total of 4646 proteins were identified using the 30 SPD gradient. FragPipe-Analyst was employed for missing values filtering, data normalisation, and missing value imputation. Peptide-wise linear models combined with empirical Bayes statistics were used for the differential expression analyses, using the Limma package from R Bioconductor 3.22 to generate a list of differentially expressed proteins and peptides for each pair-wise comparison.

#### 3.8.3. In Silico Analysis

The potential bioactivity of the peptides identified by LC-MS/MS was predicted using the PeptideRanker software program (http://distilldeep.ucd.ie/PeptideRanker/, data retrieved on 9 May 2025), which score peptides from 0 to 1. So, a higher PeptideRanker Ratio (PRR) value means a higher probability of being bioactive, which is based on the sequence of the peptides and the amino acid composition [61]. Those peptides with a PRR above 0.97 were analysed with the BIOPEP-UWM database (https://biochemia.uwm.edu.pl/biopep-uwm/, data retrieved on 9 May 2025) to check whether they were previously reported as bioactives, as well as to calculate the frequency of bioactive fragment occurrence in the protein sequence (A value) [62]. Moreover, this database was used to predict the bioactivity of potential peptide fragments. The ToxinPred software program (https://webs.iiitd.edu.in/raghava/toxinpred/, data retrieved on 9 May 2025) was used to predict the hydrophobicity of peptides based on amino acid composition [63].

### 3.9. Statistical Analysis

RStudio, the integrated development environment for R (v.4.4.1, Posit Software, PBC, Boston, MA, USA), was used for the statistical analyses of the results. A two-way analysis of variance (ANOVA) was performed to evaluate the effect of MW irradiation pre-treatment and enzymatic hydrolysis conditions on the parameters evaluated in the samples, and Tukey’s tests were run when significant differences were observed (*p* < 0.05).

## 4. Conclusions

Chicken feather hydrolysates obtained with MW irradiation and simple hydrolysis with Alcalase showed the highest antioxidant activity. MW irradiation pre-treatment increased the Neprilysin-inhibitory activity of the samples, although ACE-I- and DPP-IV-inhibitory activities were higher in those hydrolysates without MW irradiation pre-treatment. Peptide sequences of the samples C, MWC, A, and MWA were identified by LC-MS/MS and further characterised through in silico analysis, evidencing the presence of bioactive fragments within their sequences. These results confirmed that MW irradiation pre-treatment improved Alcalase hydrolysis, leading to the generation of bioactive peptides with potential multifunctional properties, including antioxidant, antidiabetic, and antihypertensive activities. This work highlights the potential of combining MW irradiation and enzymatic hydrolysis as a sustainable strategy for the revalorisation of chicken feathers, although further studies are required to explore new potential applications for this by-product.

## Figures and Tables

**Table 1:** In silico analysis of the identified peptides with PeptideRanker Score > 0.97.

Sample ^d^	Peptide Sequence	Parent Protein	PRR ^a^	Hydrophobicity	A ^b^	Bioactivity ^c^	Potential Bioactive Peptide Fragments ^c^			
							Antioxidant Peptides	ACE-I-Inhibitory Peptides	DPP-IV-Inhibitory Peptides	Neprilysin-Inhibitory Peptides
C	GLGGGLGGGLGGGLAGGSSGS	Keratin, type II cytoskeletal 5 (Positions 511–531)	0.9778	0.17	0.9048	ACE inhibitor	-	LA, GL, AG, GS, GG, SG, LG	LA, GL, AG, GG	GG
					0.6190	DPP-IV inhibitor				
					0.3333	NEP inhibitor				
	GGGGFGGGGIGGGGFGGF	Keratin, type II cytoskeletal 1 (Positions 105–122)	0.9777	0.27	1.0556	ACE inhibitor	-	GF, IG, GI, FG, GG, FGG	GF, GG, GI	FG, GG
					0.7778	DPP-IV inhibitor				
					0.6667	NEP inhibitor				
	GGGGFGGGGFGGGGIGGGGF	Keratin, type II cytoskeletal 1 (Positions 100–119)	0.9758	0.26	1.0500	ACE inhibitor	-	GF, IG, GI, FG, GG	GF, GG, GI	FG, GG
					0.8000	DPP-IV inhibitor				
					0.7000	NEP inhibitor				
	GLGGGLGGGLGGSLAGGGSGS	Keratin, type II cytoskeletal 5 (Positions 513–533)	0.9711	0.17	0.9048	ACE inhibitor	-	LA, GL, AG, GS, GG, SG, LG	LA, SL, GL, AG, GG	GG
					0.6190	DPP-IV inhibitor				
					0.3333	NEP inhibitor				
	GGGYGGGGFGGGGFGGGGF	Keratin, type II cytoskeletal 1 (Positions 91–109)	0.9711	0.22	1.1579	ACE inhibitor	-	GGY, GY, YG, GF, FG, GG, YGG, FGG	GF, GG, GY, YG	FG, GG
					0.8421	DPP-IV inhibitor				
					0.6842	NEP inhibitor				
A	PFWPGLFA	60S acidic ribosomal protein P1 (Positions 41–48)	0.9826	0.3	0.6250	ACE inhibitor	-	LF, PGL, GL, PG, PW	FA, WP, GL, PF, PG	FW
					0.6250	DPP-IV inhibitor				
					0.1250	NEP inhibitor				
	PPGPPGPPGPPSGGF	Collagen alpha-1(I) chain (Positions 1171–1185)	0.9749	0.04	0.4000	Antioxidative	GPP, GP	GP, GF, GG, SG, PG, GPP, PP	GP, PP, PPG, GF, GG, PG, PS	GG, GP
					1.0667	ACE inhibitor				
					1.0667	DPP-IV inhibitor				
					0.2667	NEP inhibitor				
	GAAFMLGF	Vitellogenin-2 (Positions 1494–1501)	0.9736	0.35	0.3750	Antioxidative	GAA, LGF, GA	AF, AA, GF, GA, LG, LGF	GA, AA, AF, GF, ML	-
					0.7500	ACE inhibitor				
					0.6250	DPP-IV inhibitor				
	GGGFGGGFGGGF	Keratin, type I cytoskeletal 9 (Positions 111–122)	0.9706	0.27	1.0833	ACE inhibitor	-	GF, FG, GG, FGG	GF, GG	FG, GG
					0.7500	DPP-IV inhibitor				
					0.6667	NEP inhibitor				
MWC	QFGFFLPL	Ephrin type-A receptor 6 (Positions 12–19)	0.9845	0.29	0.2500	Antioxidative	LPL, FGF	PL, FFL, GF, FG, LPL, FF, LP, FGF	LP, FL, LPL, PL, GF, QF	FG
					1.0000	ACE inhibitor				
					0.8750	DPP-IV inhibitor				
					0.1250	NEP inhibitor				
	FGGGGFGGGGFGGGFGG	Keratin, type I cytoskeletal 10 (Positions 117–133)	0.9726	0.27	1.1765	ACE inhibitor	-	GF, FG, GG, FGG	GF, GG	FG, GG
					0.7059	DPP-IV inhibitor				
					0.7647	NEP inhibitor				
MWA	GGLGGGLGGGLGGGLGGGLGGG	Keratin, type II cytoskeletal 5 (Positions 511–532)	0.9947	0.24	0.9545	ACE inhibitor	-	GL, GG, LG	GL, GG	GG
					0.7273	DPP-IV inhibitor				
					0.5000	NEP inhibitor				
	GSGSGYGGGLGGGLGGGLGGG	Keratin, type II cytoskeletal 5 (Positions 503–523)	0.9863	0.17	1.0000	ACE inhibitor	-	GY, YG, GL, GS, GG, SG, LG, YGG	GL, GG, GY, YG	GG
					0.6190	DPP-IV inhibitor				
					0.3810	NEP inhibitor				
	GGGSFGGGGFGGGGFGGGF	Keratin, type I cytoskeletal 10 (Positions 113–131)	0.9813	0.23	1.1053	ACE inhibitor	-	GF, FG, GS, GG, SF, FGG	GF, GG, SF	FG, GG
					0.7368	DPP-IV inhibitor				
					0.6842	NEP inhibitor				
	GRCCLEGPFWHFL	Spatacsin (Positions 76–88)	0.9793	0.02	0.3077	ACE inhibitor	-	GP, GR, EG, FW	GP, FL, EG, HF, PF, WH, GPF	FW, GP
					0.5385	DPP-IV inhibitor				
					0.1538	NEP inhibitor				
	FFYPLDF	Peroxiredoxin-2 (Positions 41–47)	0.9768	0.23	0.1429	Antioxidative	FY	FY, YP, PL, DF, FF	YP, PL, FF	-
					0.7143	ACE inhibitor				
					0.4286	DPP-IV inhibitor				
	GGFGGGGFGGGGFGGGGF	Keratin, type II cytoskeletal 1 (Positions 117–134/122–139)	0.9762	0.26	1.1111	ACE inhibitor	-	GF, FG, GG, FGG	GF, GG	FG, GG
					0.7778	DPP-IV inhibitor				
					0.7222	NEP inhibitor				
	GGGGFGGGGFGGGGIGGGGF	Keratin, type II cytoskeletal 1 (Positions 100–119)	0.9758	0.26	1.0500	ACE inhibitor	-	GF, IG, GI, FG, GG, FGG	GF, GG, GI	FG, GG
					0.8000	DPP-IV inhibitor				
					0.7000	NEP inhibitor				
	NFDMPFIF	Protein-glutamine gamma-glutamyltransferase E (Positions 383–390)	0.9755	0.17	0.3750	ACE inhibitor	-	IF, NF, DM	MP, NF, PF, MPF	-
					0.5000	DPP-IV inhibitor				
	GSGFGGFGGF	Keratin, type I cytoskeletal 9 (Positions 128–137)	0.9743	0.25	1.1000	ACE inhibitor	-	GF, FG, GS, GG, SG, FGG	GF, GG	FG, GG
					0.5000	DPP-IV inhibitor				
					0.4000	NEP inhibitor				
	GGGGFGGGGFGGGF	Keratin, type I cytoskeletal 10 (Positions 118–131)	0.9741	0.26	1.0714	ACE inhibitor	-	GF, FG, GG, FGG	GF, GG	FG, GG
					0.7857	DPP-IV inhibitor				
					0.7143	NEP inhibitor				
	GGGGFGGGGFGGGGF	Keratin, type II cytoskeletal 1 (Positions 95–109)	0.9729	0.25	1.0667	ACE inhibitor	-	GF, FG, GG, FGG	GF, GG	FG, GG
					0.8000	DPP-IV inhibitor				
					0.7333	NEP inhibitor				
	GGLGSGLGQSLGQVGGSLASLTGQIS	Thyroid receptor-interacting protein 11 (Positions 6–31)	0.9720	0.08	0.0385	Antioxidative	LT	LA, VG, GL, GS, GQ, GG, SG, LG, TG, ASL	LA, SL, GL, AS, GG, LT, QI, QS, QV, TG, VG	GG
					0.6538	ACE inhibitor				
					0.5769	DPP-IV inhibitor				
					0.0769	NEP inhibitor				
	GGGSFGGGSFGGGGFGGGGF	Keratin, type I cytoskeletal 10 (Positions 108–127)	0.9719	0.21	1.1000	ACE inhibitor	-	GF, FG, GS, GG, SF, FGG	GF, GG, SF	FG, GG
					0.7000	DPP-IV inhibitor				
					0.6500	NEP inhibitor				
	GSGFGGGSGFGGGGFGGGGF	Keratin, type II cytoskeletal 2 epidermal (Positions 97–116)	0.9719	0.21	1.1000	ACE inhibitor	-	GF, FG, GS, GG, SG, FGG	GF, GG	FG, GG
					0.6000	DPP-IV inhibitor				
					0.5500	NEP inhibitor				
	GGSGFGGGGFGGGGF	Keratin, type II cytoskeletal 2 epidermal (Positions 102–116/118–132)	0.9718	0.22	1.0667	ACE inhibitor	-	GF, FG, GS, GG, SG, FGG	GF, GG	FG, GG
					0.6667	DPP-IV inhibitor				
					0.6000	NEP inhibitor				
	GGGYGGGGFGGGGFGGGGF	Keratin, type II cytoskeletal 1 (Positions 91–109)	0.9711	0.22	1.1579	ACE inhibitor	-	GGY, GY, YG, GF, FG, GG, YGG, FGG	GF, GG, GY, YG	FG, GG
					0.8421	DPP-IV inhibitor				
					0.6842	NEP inhibitor				
	GGGFGGGGFGGGGFG	Keratin, type II cytoskeletal 1 (Positions 114–128)	0.971	0.25	1.0667	ACE inhibitor	-	GF, FG, GG, FGG	GF, GG	FG, GG
					0.7333	DPP-IV inhibitor				
					0.7333	NEP inhibitor				
	SPGGFGPGGF	Keratin, type II cytoskeletal 3 (Positions157–166)	0.9707	0.16	0.1000	Antioxidative	GP	GP, GF, FG, GG, PG	GP, SP, GF, GG, PG	FG, GG, GP
					0.8000	ACE inhibitor				
					0.8000	DPP-IV inhibitor				
					0.4000	NEP inhibitor				
	GGGGFGGGGFGGGFG	Keratin, type I cytoskeletal 10 (Positions 118–132)	0.9703	0.25	1.0667	ACE inhibitor	-	GF, FG, GG, FGG	GF, GG	FG, GG
					0.7333	DPP-IV inhibitor				
					0.7333	NEP inhibitor				
	GGGSFGGGGFGGGGF	Keratin, type I cytoskeletal 10 (Positions 113–127)	0.97	0.22	1.0667	ACE inhibitor	-	GF, FG, GS, GG, SF, FGG	GF, GG, SF	FG, GG
					0.7333	DPP-IV inhibitor				
					0.6667	NEP inhibitor				

^a^ PRR: PeptideRanker Ratio; values closer to 1 indicate higher probability to be bioactive. Only peptides with PRR > 0.97 are included. ^b^ A parameter: frequency of bioactive fragment occurrence in the protein sequence, which allows the characterisation of potential precursors of bioactive peptides according to the BIOPEP database. ^c^ Bioactivity: Potential bioactivity of the peptides according to the BIOPEP database. Only the activities evaluated in this study are included. ^d^ Samples: C: control, without microwave irradiation pre-treatment or enzymatic hydrolysis; A: simple Alcalase hydrolysis; MWC: microwave irradiation pre-treatment control, without enzymatic hydrolysis; MWA: microwave irradiation pre-treatment and simple Alcalase hydrolysis.

## Data Availability

The raw data supporting the conclusions of this article will be made available by the authors on request.

## Supplementary Materials

The following supporting information can be downloaded at: https://www.mdpi.com/article/10.3390/ijms26136344/s1.
